# Effects of Ambient O_3_ on Respiratory Mortality, Especially the Combined Effects of PM_2.5_ and O_3_

**DOI:** 10.3390/toxics11110892

**Published:** 2023-10-30

**Authors:** Ye Deng, Junlong Wang, Li Sun, Yue Wang, Jiaoyang Chen, Zhixin Zhao, Tianyun Wang, Yuting Xiang, Yuting Wang, Jiamei Chen, Miao He

**Affiliations:** 1 Liaoning Key Laboratory of Environmental Health Damage Research and Assessment, Department of Environmental Health, School of Public Health, Ministry of Education, China Medical University, Shenyang 110122, China; 2 Liaoning Provincial Center for Disease Control and Prevention, Shenyang 110005, China; 3 Key Laboratory of Environmental Stress and Chronic Disease Control & Prevention, Ministry of Education, China Medical University, Shenyang 110122, China

**Keywords:** O_3_, PM_2.5_, synergistic interaction, respiratory mortality

## Abstract

Background: In China, the increasing concentration of ozone (O_3_) has emerged as a significant air pollution issue, leading to adverse effects on public health, particularly the respiratory system. Despite the progress made in managing air pollution in China, it is crucial to address the problem of environmental O_3_ pollution at present. Methods: The connection between O_3_ exposure and respiratory mortality in Shenyang, China, from 2014 to 2018 was analyzed by a time-series generalized additive regression model (GAM) with quasi-Poisson regression. Additionally, the potential combined effects of fine particulate matter (PM_2.5_) and O_3_ were investigated using the synergy index (SI). Results: Our findings indicate that each 10 μg/m^3^ increase in O_3_ at lag 2 days was associated with a maximum relative risk (RR) of 1.0150 (95% CI: 1.0098–1.0202) for respiratory mortality in the total population. For individuals aged ≥55 years, unmarried individuals, those engaged in indoor occupations, and those with low educational attainment, each 10 μg/m^3^ increase in O_3_ at lag 07 days was linked to RR values of 1.0301 (95% CI: 1.0187–1.0417), 1.0437 (95% CI: 1.0266–1.0610), 1.0317 (95% CI: 1.0186–1.0450), and 1.0346 (95% CI: 1.0222–1.0471), respectively. Importantly, we discovered a synergistic effect of PM_2.5_ and O_3_, resulting in an SI of 2.372 on the occurrence of respiratory mortality. Conclusions: This study confirmed a positive association between O_3_ exposure and respiratory mortality. Furthermore, it highlighted the interaction between O_3_ and PM_2.5_ in exacerbating respiratory deaths.

## 1. Introduction

Globally, the COVID-19 pandemic claimed over 6.9 million lives, primarily due to respiratory illnesses [1]. However, even before the pandemic, several respiratory diseases ranked among the top 10 causes of mortality worldwide [2]. For instance, chronic respiratory diseases, affecting approximately 544.9 million people, were the third leading cause of mortality in 2017 [3], and lower respiratory tract infections held the fifth position in 2015 [4]. In China alone, respiratory diseases resulted in an estimated 3.25 million deaths in 2015 [5]. With the effects of population aging, the number of respiratory deaths is expected to steadily increase, imposing a significant burden on society. Therefore, investigating risk factors for respiratory diseases in public health is of utmost importance. Common respiratory diseases include chronic obstructive pulmonary disease (COPD), lower respiratory tract infections, asthma, occupational lung disease, and pulmonary hypertension. These diseases have numerous potential and complex causative factors, including ambient air pollution [6,7,8].

Approximately 4 million fatalities occur annually due to ambient air pollution [9]. Consequently, air pollution represents one of the most critical environmental hazards to human health [10] and has gained prominence on the global health agenda [11]. Fortunately, the implementation of environmental management measures in recent years has led to a decline in air pollution across most regions in China [12]. Nevertheless, the aging society and growing population significantly modify the group susceptible to respiratory diseases [13]. Population-weighted annual average ozone (O_3_) concentrations in China have consistently risen between 2013 and 2017, particularly in the eastern coastal regions [14]. Although age and gender have been identified as factors influencing respiratory susceptibility to O_3_ [15], the subgroups specifically sensitive to O_3_ have not been systematically studied.

Past systematic reviews and meta-analyses have revealed that inhaling fine particulate matter (PM_2.5_) can have detrimental effects on respiratory health [16,17]. It is attributed to the induction of an inflammatory response [18,19], stimulation of oxidative stress [20], and activation of immune cells [21]. Furthermore, rising levels of O_3_ in the atmosphere pose a significant challenge in controlling air pollution, leading to adverse impacts on public health [14,15]. Global O_3_-attributable mortality has increased by 46% between 2000 and 2019 [22], raising concerns about its potential negative effects on various physiological systems, including cardiovascular [23,24,25], neurological [26], and respiratory [27,28] systems. Therefore, it is crucial to comprehensively investigate both PM_2.5_ and O_3_, which are two major atmospheric toxins. Although several epidemiological studies have examined the potential synergy between PM_2.5_ and O_3_, with a specific focus on cardiovascular and nonaccidental mortality [29,30], the evidence for O_3_ and its interaction with PM_2.5_ on respiratory mortality remains unclear.

The primary objective of this research was to assess the association between exposure to O_3_ and PM_2.5_ and the potential risk of respiratory mortality. Additionally, the study aimed to elucidate the possible synergistic effect of PM_2.5_ and O_3_ on respiratory mortality.

## 2. Materials and Methods

### 2.1. Study Area

Shenyang is located in the northeast of China, with a sub-humid temperate continental climate. Here, summers are hot and wet, while winters are frigid and dry, with sufficient sunlight, strong winds, and low levels of relative humidity. The urban population of Shenyang reached 3.8 million in 2014 and was growing every year. In parallel, Shenyang has confronted severe air pollution issues in recent years due to the burning of coal for winter heating, as well as its status as one of China’s industrial centers in the past.

### 2.2. Data on Mortality Associated with Respiratory Disease

This study employed cluster sampling to gather data on respiratory disease mortality in Shenyang City, from the death registration system of the Liaoning Provincial Center for Disease Control and Prevention, between 2014 and 2018. This data recorded the personal information of each deceased person, including time of death, gender, age, nationality, marital status, work, education level, and the direct cause of death with its ICD-10 (International Classification of Diseases, 10th Revision) code. The data on fatalities for respiratory diseases (J00–J99) were analyzed. Lung cancer (C33–C34) was not included in this study, because the cause and course of lung cancer were more complex compared with common respiratory diseases. After filtering out unknown causes, abnormal records, and absent data, the number of respiratory disease fatalities was 35,385.

### 2.3. Data on Environmental Exposure

The Department of Ecology and Environment of Liaoning Province set up 11 fixed-site stations at a 2 km × 2 km spatial resolution to monitor air quality in Shenyang. Air pollutants were measured and recorded daily at the 11 stations, including particulate matter < 2.5 μm in diameter (PM_2.5_), particulate matter measuring between 2.5 and 10 μm in diameter (PM_10_), ozone (O_3_), carbon monoxide (CO), nitrogen dioxide (NO_2_) and sulfur dioxide (SO_2_). Meteorology data was also obtained from the 11 stations, which encompassed daily average temperature, barometric pressure, wind speed, and relative humidity. Air pollutants and meteorology data were quantified at a time resolution of 1 h.

### 2.4. Statistical Modeling

In this study, we used a time-series generalized additive regression model (GAM) with quasi-Poisson regression [31] to investigate the connection between air pollution and respiratory disorders in Shenyang. The model adjusted the influence of time, meteorology factors, and the holiday effect. First, a natural cubic spline function was established for temperature, relative humidity, and time variables, respectively, accounting for their potential non-linear effects on the respiratory system [32]. To streamline repetitive testing and model selection procedures, we adhered to prior model specifications and degrees of freedom (df) [24,33]. Therefore, we set a df of 7 per year for time, a df of 6 for temperature, and a df of 3 for relative humidity and incorporated weekends and official holidays into the model. Lastly, PM_2.5_ and O_3_ were included in the regression model, respectively. We proceeded with model diagnostics by evaluating the residuals of the core model.

The core model was established as follows:

Log(E[Y*_n_*]) = α + β(X*_n_*) + ns(T, df = 6) + ns(RH, df = 3)+ ns(Time, df = 7 per year) + DOW + OH

where *n* is the time of the observation, E (Y_*n*_) is the expected number of deaths from respiratory diseases, X_*n*_ is the concentration of air pollutant; β is the log-relative risk of respiratory mortality associated with a unit increment in pollutant concentration; ns ( ) represents the natural cubic spline function; T, RH and Time indicate the daily average temperature, relative humidity and time variables, respectively; DOW is the weekend effect; and OH is the official holiday effect during the study period.

To assess the lag effect of pollutants, we further added lag structures to the model [24,34]. The Lag function was used to investigate the effect of single-day lags, while the runMean function was utilized to investigate the cumulative effect of moving average lags. According to the significance of the model analysis results, the effects of O_3_ were explored at single-day lags of lag 0–lag 7 and multi-day moving average lags of lag 01–lag 07 [35]. Lag 0 is the average concentration of O_3_ on the current day and lag1 is the average concentration of O_3_ on the previous day, etc. Lag 01 is the moving average concentration of O_3_ on the present day and the previous day, and so on. Then, we discovered no short-term effect of PM_2.5_ on respiratory mortality at lag 0–lag 7 or lag 01–lag 07 (Figure S3). Previous studies suggested that the dominant effects of PM_2.5_ on death could be observed at long-term exposure [36,37]. Accordingly, we extended PM_2.5_ multi-day cumulative lag days to 50 days. To investigate potential non-linear correlations, we also displayed the exposure-response curves for the links between pollutants and respiratory illness mortality [38].

Next, we conducted a stratified study of O_3_ between the cold season (November to March) and the warm season (April to October) due to a significant confounding factor: temperature [39]. Moreover, to identify potentially susceptible subgroups with a higher risk of O_3_ exposure, stratified analyses were performed based on several socioeconomic and personal factors, including age, gender, nationality, marital status, job, and level of education (high education is junior high school and above; low education is below junior high school). To identify potential subtypes of respiratory diseases with a higher risk of O_3_ exposure, stratified analyses were performed based on specific respiratory disease categories, including influenza and pneumonia, lower respiratory tract infection disease, chronic lower respiratory disease, and respiratory failure.

Spearman’s rank correlation was then used to analyze the association between atmospheric pollutants and meteorology factors. Finally, we performed sensitivity analyses to assess the robustness of our results. Dual-pollutant and multi-pollutant models were set up to evaluate the robustness of the association between O_3_ and respiratory mortality [28].

### 2.5. Interaction Analysis

The study further conducted a comprehensive analysis of the potential association between PM_2.5_ and O_3_, applying both multiplicative and additive interaction models. To assess multiplicative interactions, a product term of PM_2.5_ and O_3_ was included in the generalized additive model. The synergy index (SI) was used to assess addictive interactions [40,41]. An SI value of >1 indicates a cooperative interaction, implying that the combined impacts of PM_2.5_ and O_3_ were greater than the total of their individual effects. On the contrary, it suggests an antagonist effect when an SI value is <1.

O_3_ was divided into two levels, namely low and high, based on a cut-point of 100 μg/m^3^, as required by the Grade II national standards for O_3_ concentration. PM_2.5_ was also divided into two levels, based on a cut-point of 71 μg/m^3^—the 75th percentile value of PM_2.5_ concentrations from the obtained data. Subsequently, the combination of these two variables was represented by a novel variable that consisted of the following four classifications: (1) low PM_2.5_ and low O_3_; (2) low PM_2.5_ and high O_3_; (3) high PM_2.5_ and low O_3_; and (4) high PM_2.5_ and high O_3_.

We conducted all statistical analyses using R, version 4.3.0. Effect estimates were presented as relative risks (RR) with 95% confidence intervals (CIs). A two-sided *p* value of <0.05 was considered statistically significant.

## 3. Results

### 3.1. Characteristics of the Study Population

Table 1 presents the characteristics of the participants included in the research. The study encompassed 35,385 deaths from respiratory diseases. Approximately 90% of the deaths were aged 55 years and older. Only 8148 individuals, accounting for 23.03% of the total deaths, had received education of junior high school and higher. The distribution of the respiratory deaths is revealed in Table S1.

### 3.2. Distribution of Air Pollutants

Table 2 shows the statistical characteristics of the environmental meteorology factors and air quality. Here, meteorology and air quality data were collected from 2014 to 2018, in Shenyang. Meanwhile, a total of 35,385 people died from respiratory diseases over the five-year period. The average concentration of NO_2_ is higher than the Grade II national standards for air quality (40 μg/m^3^); the median and average concentrations of both PM_2.5_ and PM_10_ are also higher than the Grade II national standards for air quality (35 and 70 μg/m^3^). Table S2 and Figure S1 show the variation in pollutant exposure. The annual average concentration of the six pollutants (O_3_, PM_2.5_, PM_10_, SO_2_, NO_2_, CO) reached their highest levels in 2014. NO_2_ concentration has exhibited a gradual decline. Furthermore, it is worth noting that a cyclic variation can be observed for concentration changes of both O_3_ and PM_2.5_. However, PM_2.5_ reaches a maximum in winter and O_3_ reaches its maximum in summer of every year (Figure S1). With regard to meteorology factors, the mean temperature was 9.1 °C and the mean relative humidity was 59.9% (Table 2).

Table 3 indicates that there is a positive correlation among the five pollutants (PM_2.5_, PM_10_, SO_2_, NO_2_, and CO), particularly between PM_2.5_ and PM_10_ (*r* = 0.90). Nevertheless, the five pollutants mentioned are negatively correlated with O_3,_ and temperature is positively correlated with O_3_ (*r* = 0.70).

### 3.3. Air Pollution Exposure and Respiratory Mortality

**Figure 1:**
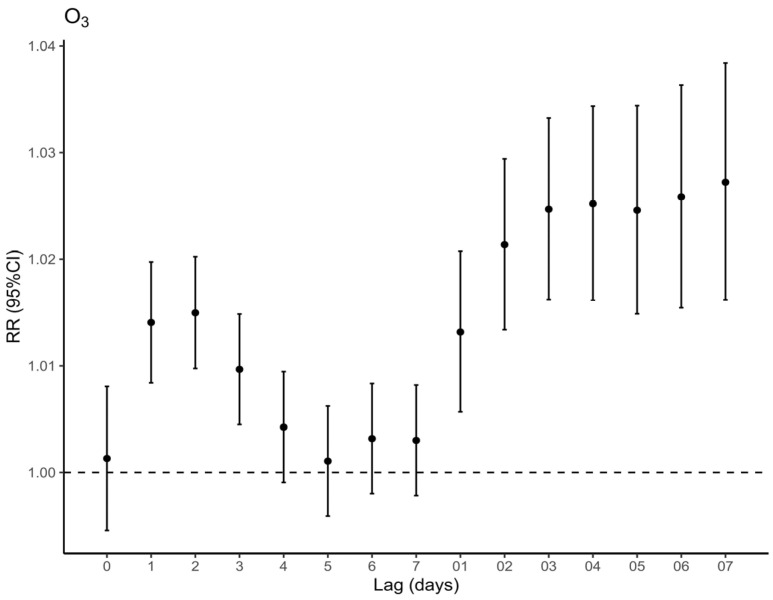
Relative risks of respiratory death per 10 μg/m^3^ increase in O_3_ concentration at single-day lags and multi-day cumulative lags.

**Figure 2:**
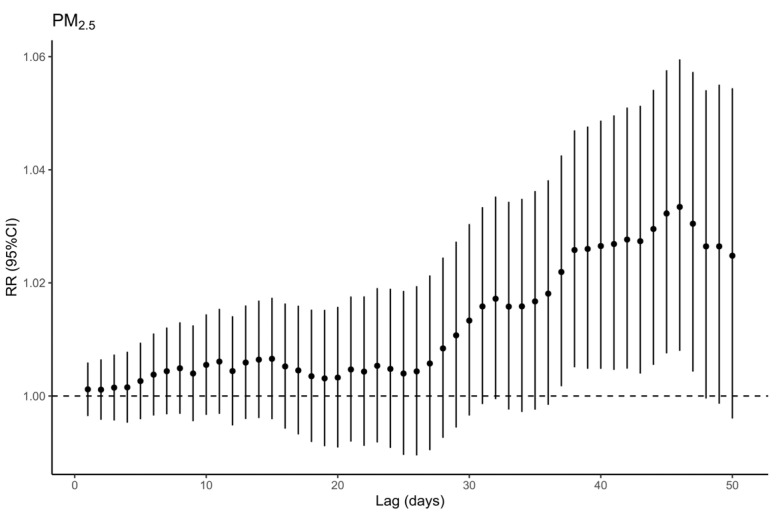
Relative risks of respiratory death per 10 μg/m^3^ increase in PM_2.5_ concentration at multi-day cumulative lags.

Figure 1 shows the impact of O_3_ on total respiratory deaths on the day of non-lag, single-day lags of 1–7 days, and multi-day moving average lags of 01–07 days (expressed as RR and its 95% CI). The greatest impact of O_3_ was observed at lag 2 of all single-day lags, with RR = 1.0150 (95% CI: 1.0098–1.0202). As for the moving average lags, the effect of O_3_ grows slowly from lag 01 to lag 07, reaching a maximum at lag 07, with RR = 1.0272 (95% CI: 1.0162–1.0384). Notably, O_3_ exposure significantly elevates the risk of respiratory fatalities during the warm season, while no statistically significant association has been observed during the cold season (Figure S2). Meanwhile, we discovered no effect of short-term exposure to PM_2.5_ (Figure S3) but a significant effect of long-term exposure to PM_2.5_ at a moving average lag of 46 days, with RR = 1.0351 (95% CI: 1.0095–1.0613) (Figure 2).

**Figure 3:**
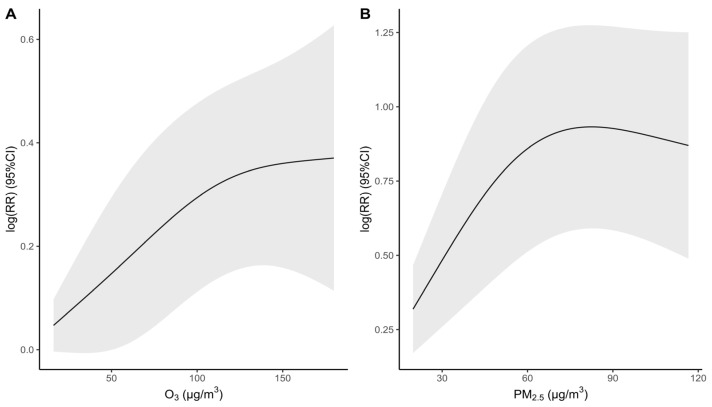
Exposure-response curves between the air pollutant and respiratory death risk ((**A**)—O_3_ concentration: moving an average lag of 7 days; (**B**)—PM_2.5_ concentration: moving an average lag of 46 days).

Figure 3 indicates that the respiratory mortality risk is augmented with O_3_ and PM_2.5_ concentrations. A safety threshold of O_3_ concentration at 52 μg/m^3^ for respiratory mortality was observed. There was no safety threshold in the exposure-response relationships between PM_2.5_ and respiratory mortality.

### 3.4. Joint Effects of Air Pollutants on Respiratory Mortality

Table 4 describes the interactions between O_3_ and PM_2.5_ on the mortality rate of respiratory illnesses. The individual effect of PM_2.5_ is 0.978 (95% CI: 0.945–1.013), the individual effect of O_3_ is 1.065 (95% CI: 1.017–1.116), and their cooperative effect is 1.102 (95% CI: 1.015–1.196). Despite the 95% CI for the relative excess risk due to interaction (RERI) and attributable proportion of interaction (AP) encompassing a value of 0, it is noteworthy that the synergy index (SI) is a statistically significant value of 2.372 (95% CI: 1.127–3.617). The interaction item added to the model failed to attain statistical significance in the multiplicative model (*p* > 0.05).

### 3.5. Subgroup Analysis of O_3_ Exposure

**Figure 4:**
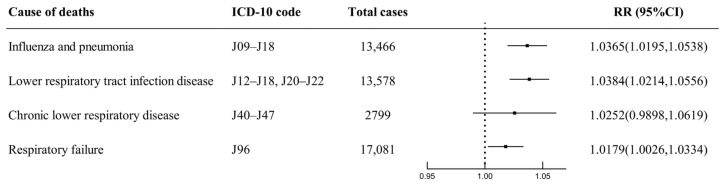
Relative risks of the cause-specific respiratory mortality per 10 μg/m^3^ increase in O_3_ concentration of moving average lag 07.

Figure 4 shows the impact of O_3_ on respiratory mortality after stratification according to specific respiratory disease categories, including influenza and pneumonia, lower respiratory tract infection disease, chronic lower respiratory disease, and respiratory failure. Per 10 μg/m^3^ increase of O_3_ at lag 07 days is associated with 1.0365 (95% CI: 1.0195–1.0538), 1.0384 (95% CI: 1.0214–1.0556), 1.0252 (95% CI: 0.9898–1.0619), and 1.0179 (95% CI: 1.0026–1.0334) RR of death from influenza and pneumonia, lower respiratory tract infection disease, chronic lower respiratory disease, and respiratory failure.

**Figure 5:**
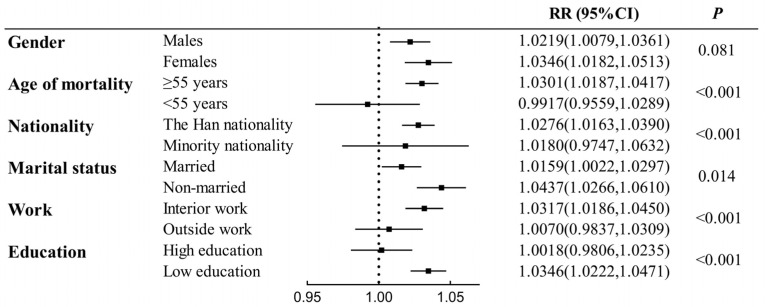
Relative risks of the respiratory mortality per 10 μg/m^3^ increase in O_3_ concentration of moving average lag 07 for the population stratifications.

Figure 5 and Figure S4 show the impact of O_3_ on respiratory mortality after stratification according to personal characteristics and social factors of the population, including age, gender, nationality, marital status, job, and level of education (high education is junior high school and above; low education is below junior high school). Per 10 μg/m^3^ increase of O_3_ at lag 07 is associated with 1.0346 (95% CI: 1.0182–1.0513), 1.0301 (95% CI: 1.0187–1.0417), 1.0276 (95% CI: 1.0163–1.0390), 1.0437 (95% CI: 1.0266–1.0610), 1.0317 (95% CI: 1.0186–1.0450), and 1.0346 (95% CI: 1.0222–1.0471) RR of respiratory mortality in females, the elderly (≥55 years old), the Han nationality, non-married individuals, those engaged in indoor occupations, and those with low educational attainment. Figure 5 shows statistically significant differences in age groups, nationality groups, marriage groups, work groups, and education groups, but not in gender groups.

### 3.6. Sensitivity Analyses

The sensitivity analyses indicate that the findings obtained from the primary models are robust. When including one or several pollutants in the models for modification, the effects of O_3_ remain statistically significant. With more pollutants included in the model, the effects of O_3_ became slightly greater at both lag 2 and lag 07, as shown in Table 5.

## 4. Discussion

This study revealed a significant association between ambient O_3_ and respiratory mortality among residents, specifically influenza and pneumonia, and lower respiratory tract infection disease. The research presented a methodology for assessing the impact of O_3_ on respiratory mortality, particularly the interaction between PM_2.5_ and O_3_, in Shenyang, China from 2014 to 2018. The exposure-response curve indicated a safety threshold for O_3_ concentrations at 52 μg/m^3^ regarding respiratory mortality. With every 10 μg/m^3^ increase in O_3_ at lag 2 days, the highest RR for respiratory mortality was 1.0150 (95% CI: 1.0098–1.0202). Furthermore, by analyzing O_3_ concentrations of the moving average lag, the cumulative exposure to O_3_ was found to have an even more severe impact. Moreover, certain subpopulations including unmarried individuals, those engaged in indoor occupations, and those with low educational attainment were identified to be more vulnerable to O_3_. Significantly, a synergistic interaction between PM_2.5_ and O_3_ regarding the impact of respiratory death was discovered, with an SI of 2.372.

### 4.1. O_3_ Exposure and Respiratory Diseases

Previous animal research has demonstrated that O_3_ exposure can increase the production of Th2 cytokines, eosinophilic airway inflammation [42], and IL-33 airway hyperresponsiveness in a dose-dependent manner [43]. Additionally, inhalation of O_3_ leads to oxidative damage due to the generation of reactive oxygen species (ROS), resulting in excessive mitochondrial oxidative stress [44]. O_3_ can also activate adrenergic and glucocorticoid receptors, causing the release of epinephrine and corticosterone into the circulation, thereby exacerbating O_3_-induced pulmonary damage and inflammation [45]. Transcriptomics studies also suggest the dysregulation of numerous pathways after O_3_ exposure, such as mitochondrial dysfunction and glucocorticoid receptor signaling [46]. Furthermore, several epidemiological studies have shown that long-term exposure to ambient O_3_ significantly contributes to respiratory mortality [14,47], particularly in areas with high O_3_ concentrations [39]. The above evidence suggests that O_3_ exposure can induce respiratory dysfunction, reinforcing our conclusions.

There has been no conclusive evidence regarding the safe threshold for acute O_3_ damage to human health [48]. A nationwide epidemiological study in China suggested that the safety threshold for O_3_’s effect on total mortality may range between 60 and 100 μg/m^3^, depending on the cause of death [15]. In a London study, a threshold O_3_ concentration of 50 μg/m^3^ was found for respiratory mortality [49], which closely aligns with our results. Another nationwide study involving 53 million U.S. Medicare beneficiaries found no evidence of a safety threshold for the effect of PM_2.5_ on respiratory mortality risk [50].

Moreover, previous studies have extensively examined the delayed effects of ambient pollutants on public health. The highest elevated risk of respiratory death from O_3_ exposure was reported as 0.78% (95% CI: 0.33%–1.24%) at lag 3 in Guangzhou, China [33], and 1.04% (95% CI: 0.04%–1.68%) at lag 0 in Nanchang, China [35]. However, our study revealed that the greatest elevated risk of respiratory death from O_3_ exposure was 1.50% (95% CI: 0.98%–2.02%) at lag 2 in Shenyang, China, calculated using an RR of 1.0150 (95% CI: 1.0098–1.0202). This suggests that the effect of O_3_ on respiratory death in Shenyang may be slightly higher than in the two southern cities of China (Guangzhou and Nanchang). Nonetheless, these variations are reasonable because population-weighted average O_3_ concentrations are different, and attributable per capita respiratory mortality rates vary across regions in China [14]. Furthermore, a few studies have confirmed our hypothesis that the cumulative effect of O_3_ on respiratory health increases over time [28,35]. In comparison to PM_2.5_, studies in mice have shown that O_3_ has a stronger potency in causing respiratory changes, possibly due to its nature as an irritant gas [51]. This observation may explain why, in our study, the optimal lag time for PM_2.5_ effects is significantly longer than for O_3_.

Our study found that influenza and pneumonia (RR = 1.0365, 95% CI: 1.0195–1.0538), as well as lower respiratory tract infection disease (RR = 1.0384, 95% CI: 1.0214–1.0556), could be more sensitive to O_3_ compared to total respiratory disease (RR = 1.0272, 95% CI: 1.0162–1.0384) at lag 07. Mice exposed to O_3_ are more susceptible to respiratory bacterial infections, partially due to toxicological interactions between bacterial lipopolysaccharide and O_3_ [52]. Additionally, O_3_ causes an increase in susceptibility to influenza A viruses by regulating the airway balance of protease/antiprotease [53]. O_3_, being a gas with the ability to deeply infiltrate the lower respiratory tract, effectively penetrates deep into the lungs [54]. A study conducted in Guangzhou showed that chronic lower respiratory diseases are more sensitive to O_3_ exposure [33]. However, our study had too few deaths from chronic lower respiratory diseases to draw definitive conclusions. The discrepancies in estimates of O_3_ effects may be attributed to variations in ambient O_3_ pollution, regional differences, population susceptibility, and levels of healthcare available.

### 4.2. Subgroups More Sensitive to O_3_

Age-related changes in the innate immune system coincide with age-related deficiencies in T-cell and B-cell function [55]. Furthermore, older individuals, often with underlying health conditions, experience prolonged exposure to ambient pollutants compared to younger individuals. These findings indicate that ambient pollutants pose greater risks to the health of the elderly population. A study conducted on healthy older adults revealed that exposure to near-ambient O_3_ can lead to impaired pulmonary function, airway damage, and inflammation [56].

Asthma emergency room visits among females aged 40–64 were more strongly associated with O_3_ exposure compared to males of the same age group, showing a higher RR of 1.21 (95% CI = 1.05–1.39) [57]. This suggests that females are more susceptible to inhaled O_3_, possibly due to estrogen levels and varying regulation of pulmonary immune function [58]. Moreover, gender-specific miRNA regulation of inflammatory gene expression may mediate different effects of pollution on health based on gender [59].

In our study, the unmarried status predominantly referred to divorced or widowed individuals, with a mean age of 75.97 years. This indicates that they have experienced the loss of a partner, creating significant psychosocial stressors. Psychosocial stressors, particularly social isolation, impact the neurological and endocrine systems and trigger detrimental metabolic and inflammatory responses. These responses are further exacerbated by O_3_ exposure, leading to increased neutrophils and IL-6 levels in lavage fluid [60]. Additionally, inhalation of O_3_ might have implications for mental health [61]. Consequently, there may be a harmful cycle involving O_3_ exposure, heightened psychological tension, and respiratory injury. There is no direct data in our study to support these speculations and further research is required.

In our investigation, the majority of outdoor workers were farmers or laborers engaged in manual labor in open-air environments for prolonged periods. Exercise-induced transition from nasal to oral respiration can render the distal lung more susceptible to O_3_ damage due to increased O_3_ exposure [62]. Surprisingly, our findings suggest that indoor employees are more sensitive to O_3_. Although most indoor O_3_ originates from outdoor sources through ventilation air, indoor emission sources can significantly elevate indoor O_3_ levels [63]. Furthermore, the work recorded in our research data refers to participants’ occupations at the time of their mortality. Since most individuals were retired and engaged in indoor work during this period, it becomes difficult to accurately determine their lifetime occupational history. Consequently, the unexpected finding of an increased risk among indoor workers may be attributed not only to indoor sources of O_3_ but also to potential misclassification of differential exposure.

In China, a 10 μg/m^3^ increase in PM_2.5_ was associated with a higher prevalence of major cardiovascular disease in participants with lower education levels but not in the well-educated population [64]. Another epidemiological study similarly demonstrated that the association between O_3_ and years of life lost was more pronounced in individuals with less education [65]. These two studies align with our findings and can be attributed to several reasons. Individuals with lower education levels tend to have poorer economic conditions in China, often residing in areas with severe air pollution [66,67]. As a result, individuals with limited education may experience higher levels of O_3_ exposure and greater susceptibility to respiratory diseases.

### 4.3. Combined Effects between PM_2.5_ and O_3_

Our results revealed an SI value of 2.372, indicating that PM_2.5_ and O_3_ have combined effects on respiratory mortality that exceed the sum of their individual impacts. However, the precise latent cellular molecular pathway responsible for these effects remains largely unidentified. Nevertheless, our results can be explained by several studies that focus on the biological mechanisms underlying the interaction between PM_2.5_ and O_3_ in relation to other health outcomes. It is plausible that similar pathogenic mechanisms, such as inflammation, oxidative stress, and cytokine induction, contribute to the observed impacts of both substances [68]. Additionally, the chemical reactions occurring on the particle surface promote interactions between O_3_ and PM_2.5_ [69]. For instance, a study investigating the interaction between O_3_ and ultrafine carbon demonstrated that combined exposure led to a greater decline in respiratory function compared to individual exposure. This finding was associated with the induction of the IL-13 pathway, increased mucin production, and interferon gene expression [70]. Moreover, higher levels of O_3_ may enhance particle reactivity [71], reduce the removal of PM_2.5_, and increase its accumulation and retention [72], thereby exacerbating its negative impact on the respiratory system.

### 4.4. Strengths and Limitations

There were several strengths in this time-series epidemiological research. Firstly, the underreporting or duplication of data was minimal due to the utilization of official sources for meteorological elements, air quality measurements, and respiratory mortality data. These sources include the Department of Ecology and Environment of Liaoning Province and the death registration system of the Liaoning Provincial Center for Disease Control and Prevention. Secondly, our study focused on subgroups particularly sensitive to O_3_, who were more likely to develop and die from respiratory illnesses. Thus, our findings offer novel perspectives on strategies to manage the global burden of respiratory illnesses in the future. Lastly, our research contributed to the emerging focus on the interaction between O_3_ and other air pollutants, highlighting a synergistic relationship between PM_2.5_ and O_3_ in terms of their impact on respiratory diseases, an aspect that has received limited attention in previous studies.

However, there were also some limitations in this study. Firstly, the ambient O_3_ concentrations were derived from fixed-site monitors rather than individual measurements, which may result in non-differential exposure misclassification errors and an underestimation of the impact of air pollutants. Secondly, the findings of this research may lack generalizability due to the methodology of cluster sampling and the specific research region (Shenyang City). Thirdly, the model did not include several unavailable confounding factors such as smoking status and medication usage. Finally, because of the nature of the ecological study design, it is not possible to establish a causal link.

## 5. Conclusions

This study confirmed a positive association between O_3_ exposure and respiratory mortality. Furthermore, we observed combined effects between O_3_ and PM_2.5_ on respiratory mortality. Our findings complemented limited previous studies by identifying subpopulations that exhibited increased sensitivity to O_3_. These findings will assist policymakers in improving the management of air pollution in the future. Moving forward, it is crucial to prioritize research on the combined effects of multiple ambient pollutants and the protection of sensitive groups in order to improve public health.

## Figures and Tables

**Table 1:** Characteristics of the respiratory deaths in Shenyang, China, during 2014–2018.

Characteristic	n (%)
Total	35,385 (100.00)
Gender	
Males	20,572 (58.14)
Females	14,813 (41.86)
Age of mortality	
≥55 years	32,780 (92.64)
<55 years	2605 (7.36)
Nationality	
The Han nationality	33,465 (94.57)
Minority nationality	1911 (5.40)
unknown	9 (0.03)
Marital status	
Married	20,932 (59.16)
Non-married	14,196 (40.12)
unknown	257 (0.73)
Work	
Interior work	24,374 (68.89)
Outside work	6532 (18.46)
unknown	4479 (12.66)
Education	
High education	8148 (23.03)
Low education	27,237 (76.97)
Cause of deaths	
Influenza and pneumonia (J09–J18)	13,466 (38.06)
Lower respiratory tract infection disease (J12–J18, J20–J22)	13,578 (38.37)
Chronic lower respiratory disease (J40–J47)	2799 (7.91)
Respiratory failure (J96)	17,081 (48.27)

**Table 2:** Distribution of meteorological elements and concentrations of air pollutants.

	Mean	Min	P25	P50	P75	Max
T (℃)	9.1	−22.8	−2.8	11.0	21.2	32.4
RH (%)	59.9	15.1	47.8	61.3	72.0	98.0
O_3_ (μg/m^3^)	66.7	9.0	35.0	59.0	88.0	250.0
PM_2.5_ (μg/m^3^)	56.1	4.0	28.0	43.0	72.0	291.0
PM_10_ (μg/m^3^)	94.6	8.0	56.0	81.0	119.0	396.0
SO_2_ (μg/m^3^)	47.5	3.0	15.0	26.0	57.0	332.0
NO_2_ (μg/m^3^)	41.8	12.0	29.0	39.0	51.0	125.0
CO (mg/m^3^)	1.0	0.3	0.7	0.9	1.2	3.3

**Table 3:** Spearman’s rank correlation coefficients between pollutants and meteorological variables.

	O_3_	PM_2.5_	PM_10_	SO_2_	NO_2_	CO	T	RH
O_3_ (μg/m^3^)	1.00							
PM_2.5_ (μg/m^3^)	−0.17 *	1.00						
PM_10_ (μg/m^3^)	−0.09 *	0.90 *	1.00					
SO_2_ (μg/m^3^)	−0.39 *	0.70 *	0.67 *	1.00				
NO2 (μg/m3)	−0.34 *	0.74 *	0.68 *	0.71 *	1.00			
CO (mg/m3)	−0.19 *	0.80 *	0.70 *	0.63 *	0.72 *	1.00		
T (℃)	0.70 *	−0.37 *	−0.35 *	−0.67 *	−0.40 *	−0.21 *	1.00	
RH (%)	−0.07 *	−0.01	−0.20 *	−0.21 *	−0.02	0.18 *	0.32 *	1.00

* *p* < 0.01.

**Table 4:** The interactions between O_3_ and PM_2.5_ on respiratory mortality.

Category	Value & 95% CI
OR	
low O_3_ + low PM_2.5_	1
high O_3_ + low PM_2.5_	1.065 (1.017, 1.116)
low O_3_ + high PM_2.5_	0.978 (0.945, 1.013)
high O_3_ + high PM_2.5_	1.102 (1.015, 1.196)
SI	2.372 (1.127, 3.617)
RERI	0.059 (−0.021, 0.139)
AP	0.054 (−0.015, 0.123)

**Table 5:** Relative risk of respiratory mortality per 10 μg/m^3^ increase in O_3_ concentration in single-pollutant, double-pollutant, and multi-pollutant models.

Pollutant(s)	Lag 2	Lag 07
O_3_	1.0150 (1.0098, 1.0202)	1.0272 (1.0162, 1.0384)
O_3_ + PM_2.5_	1.0150 (1.0098, 1.0203)	1.0273 (1.0162, 1.0385)
O_3_ + PM_10_	1.0149 (1.0097, 1.0201)	1.0269 (1.0159, 1.0381)
O_3_ + SO_2_	1.0150 (1.0098, 1.0203)	1.0276 (1.0165, 1.0388)
O_3_ + NO_2_	1.0152 (1.0099, 1.0205)	1.0278 (1.0166, 1.0391)
O_3_ + PM _2.5_ + SO_2_	1.0150 (1.0098, 1.0203)	1.0275 (1.0164, 1.0388)
O_3_ + PM _2.5_ + NO_2_	1.0152 (1.0100, 1.0205)	1.0279 (1.0167, 1.0392)
O_3_ + SO_2_ + NO_2_	1.0152 (1.0099, 1.0205)	1.0278 (1.0166, 1.0391)
O_3_ + PM _2.5_ + SO_2_ + NO_2_	1.0152 (1.0100, 1.0205)	1.0279 (1.0167, 1.0392)

## Data Availability

The data presented in this study are available on request from the corresponding author.

## Supplementary Materials

The following supporting information can be downloaded at: https://www.mdpi.com/article/10.3390/toxics11110892/s1, Table S1: Distribution of respiratory deaths; Table S2: Distribution of annual air pollutants between 2014 to 2018; Figure S1: Time series of the concentrations of NO_2_, O_3_, and PM_2.5_; Figure S2: Relative risk of respiratory death per 10 μg /m^3^ increase in O_3_ concentration at single-day lags and multi-day cumulative lags. A: the warm season (from April to October); B: the cold season (from October to April); Figure S3: Relative risk of respiratory death per 10 μg/m^3^ increase in PM_2.5_ concentration at single-day lags and multi-day cumulative lags; Figure S4: Relative risk of respiratory death per 10 μg/m^3^ increase in O_3_ concentration of population stratifications at single-day lags and multi-day cumulative lags.
