# Supplementary material for: Effects of Ambient O3 on Respiratory Mortality, Especially the Combined Effects of PM2.5 and O3
Source: Toxics. 2023 Oct 30;11(11):892. doi: 10.3390/toxics11110892 (PMC10675328; doi:10.3390/toxics11110892)
Supplement: Supplementary file 1 [file toxics-11-00892-s001.zip › toxics-2613336-supplementary.pdf]

toxics MDPI

Supplementary materials

Table S1. Distribution of respiratory deaths.

	Mean	Min	P25	P50	P75	Max
Respiratory death toll						
All	19.4	3.0	15.0	19.0	23.0	73.0
Males	11.3	1.0	8.0	11.0	14.0	35.0
Females	8.1	0.0	6.0	8.0	10.0	38.0
≥55 years	18.0	3.0	14.0	18.0	21.0	68.0
<55 years	1.4	0.0	0.0	1.0	2.0	8.0
Han nationality	18.3	2.0	14.0	18.0	22.0	70.0
Minority nationality	1.0	0.0	0.0	1.0	2.0	7.0
Married	11.5	0.0	8.0	11.0	14.0	34.0
Other marital status	7.8	0.0	5.0	7.0	10.0	39.0
Inside work	13.4	0.0	10.0	13.0	17.0	50.0
Outside work	3.6	0.0	2.0	3.0	5.0	12.0
High education	4.5	0.0	3.0	4.0	6.0	15.0
Low education	14.9	2.0	11.0	14.0	18.0	61.0

Table S2. Distribution of annual air pollutants between 2014 to 2018.

Pollutants	Year	Mean	Min	P25	P50	P75	Max
O_3 (μg/m^3)	2014	95.72	12.00	57.00	91.00	129.00	250.00
	2015	56.20	9.00	31.00	49.00	77.00	178.00
	2016	58.34	11.00	33.00	55.50	78.00	162.00
	2017	63.23	10.00	35.00	58.00	81.00	218.00
	2018	60.15	9.00	31.00	53.00	82.00	195.00
$PM_{2.5}$ (μg/m^3)	2014	71.12	12.00	40.00	59.00	91.00	277.00
	2015	66.76	10.00	31.00	51.00	86.00	291.00
	2016	52.17	4.00	30.00	43.00	64.75	221.00
	2017	48.43	7.00	25.00	36.00	60.00	224.00
	2018	42.21	6.00	21.00	34.00	55.00	186.00
PM_{10} (μg/m^3)	2014	118.78	30.00	76.00	106.00	152.00	369.00
	2015	106.54	19.00	58.00	91.00	137.00	396.00
	2016	90.57	8.00	58.25	79.00	109.00	355.00
	2017	84.53	17.00	50.00	72.00	104.00	348.00
	2018	72.33	18.00	45.00	67.00	91.00	195.00
SO_2 (μg/m^3)	2014	77.92	4.00	24.00	44.00	115.00	332.00
	2015	59.60	3.00	14.00	29.00	87.00	316.00
	2016	42.75	4.00	14.25	27.00	68.50	173.00
	2017	33.58	4.00	14.00	23.00	43.00	160.00
	2018	23.43	6.00	14.00	21.00	29.00	98.00
NO_2 (μg/m^3)	2014	51.48	18.00	37.00	48.00	62.00	116.00
	2015	44.86	18.00	31.00	40.00	55.00	125.00
	2016	38.12	13.00	27.00	36.00	46.75	89.00
	2017	39.01	14.00	28.00	35.00	49.00	109.00
	2018	35.70	12.00	25.00	33.00	45.00	78.00
CO (mg/m^3)	2014	1.09	0.30	0.80	1.00	1.40	3.30
	2015	1.02	0.32	0.64	0.86	1.22	3.12
	2016	0.92	0.31	0.66	0.86	1.10	2.59
	2017	0.95	0.31	0.62	0.83	1.16	2.69
	2018	0.97	0.38	0.69	0.87	1.18	2.35

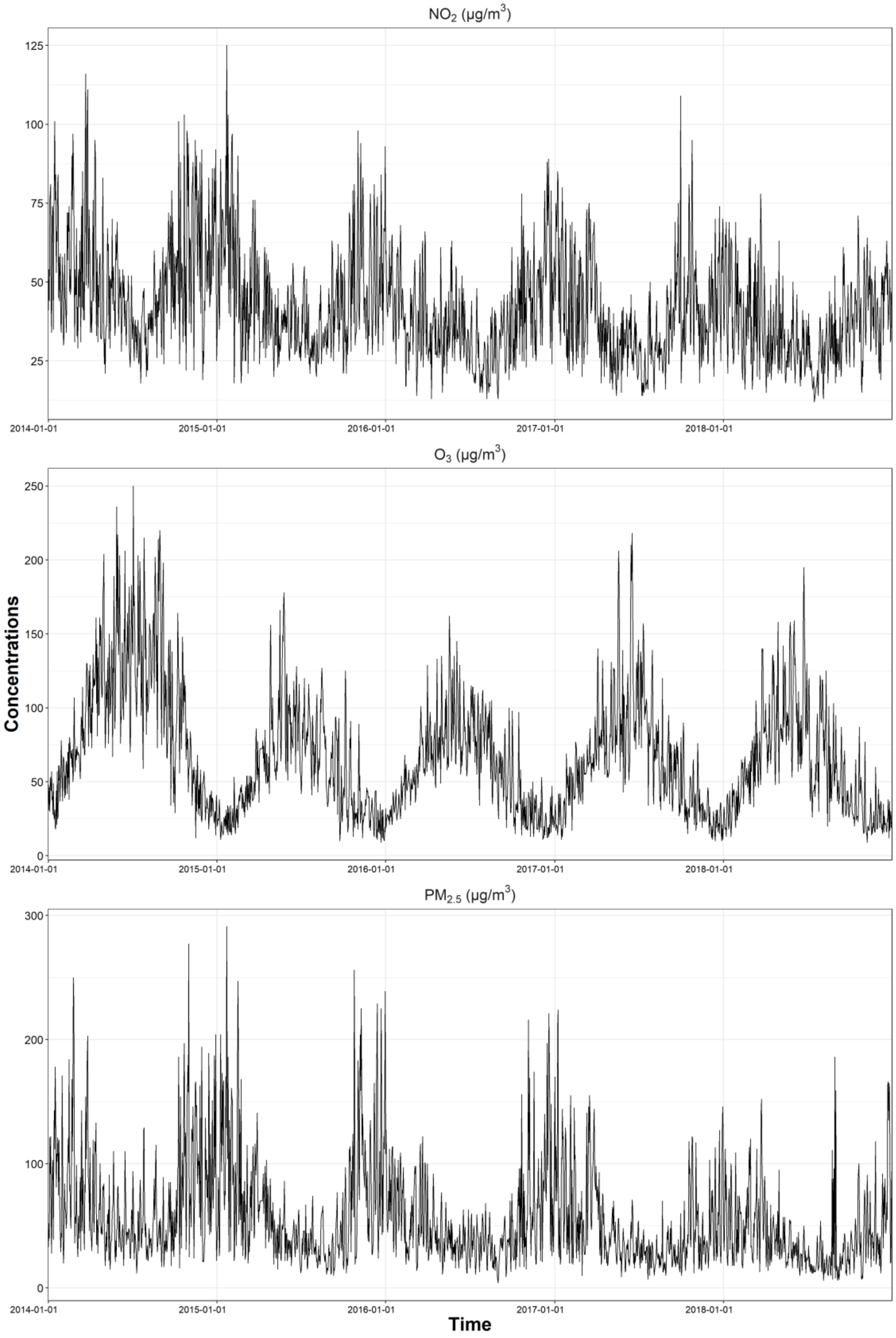

Figure S1. Time series of the concentrations of NO_2, O_3, and $PM_{2.5}$.

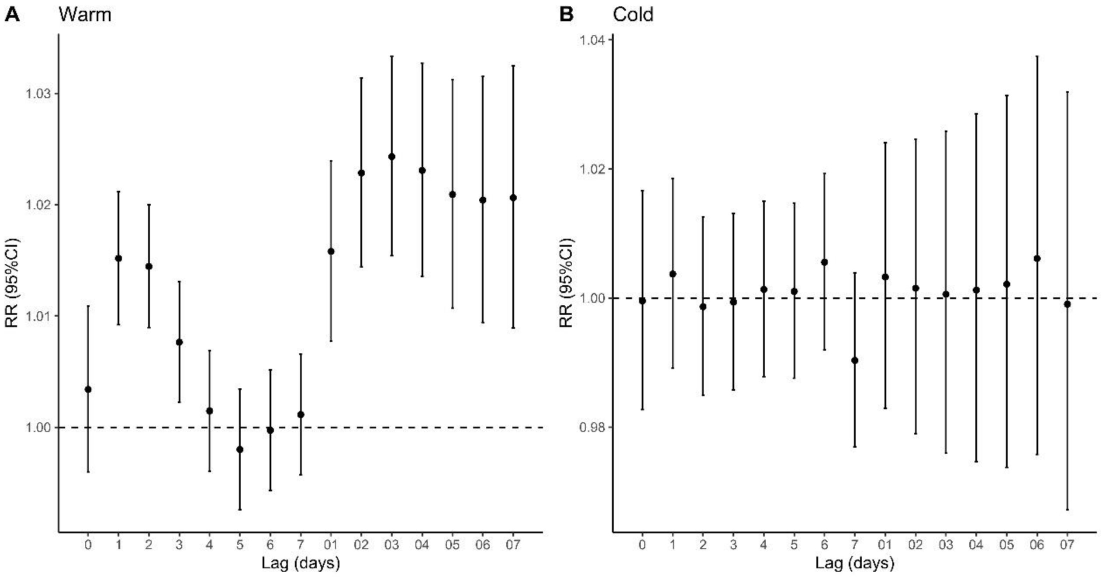

Figure S2. Relative risk of respiratory death per 10 µg /m³ increase in O_3 concentration at single-day lags and multi-day cumulative lags. A: the warm season (from April to October); B: the cold season (from October to April);.

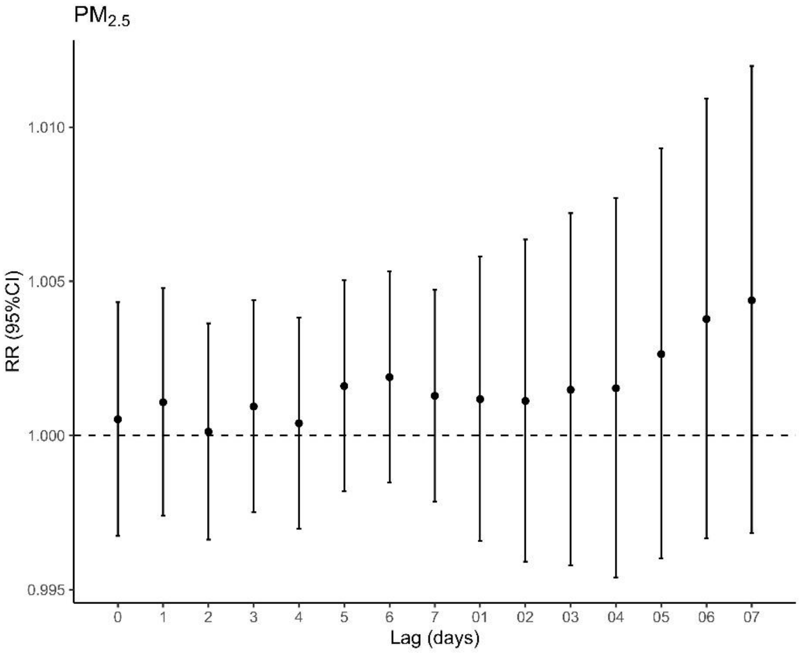

Figure S3. Relative risk of respiratory death per 10 µg/m³ increase in $PM_{2.5}$ concentration at single-day lags and multi-day cumulative lags.

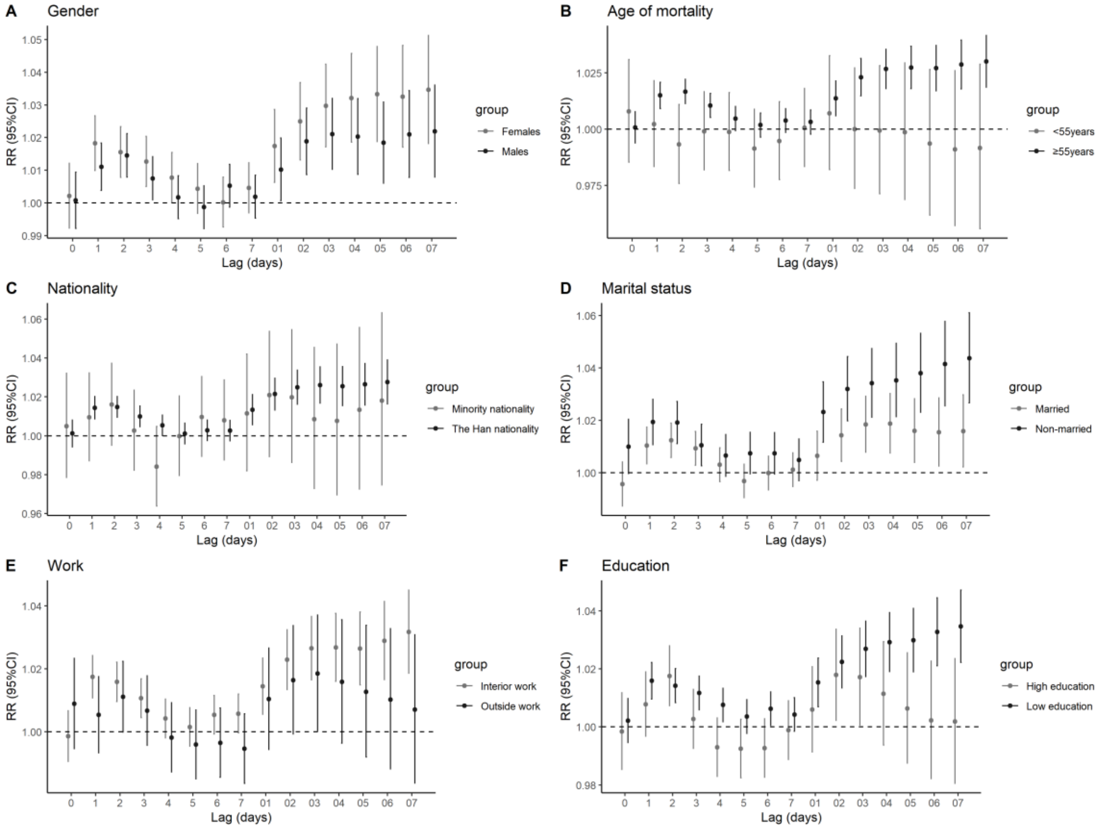

Figure S4. Relative risk of respiratory death per 10 μg/m^3 increase in O_3 concentration of population stratifications at single-day lags and multi-day cumulative lags.
